# Physicians' norms and attitudes towards substance use in colleague physicians: A cross-sectional survey in the Netherlands

**DOI:** 10.1371/journal.pone.0231084

**Published:** 2020-04-03

**Authors:** Pauline Geuijen, Marlies de Rond, Joanneke Kuppens, Femke Atsma, Aart Schene, Hein de Haan, Cornelis de Jong, Arnt Schellekens

**Affiliations:** 1 Department of Psychiatry, Donders Institute for Brain, Cognition and Behaviour, Radboud University Medical Center, Nijmegen, the Netherlands; 2 Nijmegen Institute for Scientist-Practitioners in Addiction (NISPA), Nijmegen, the Netherlands; 3 Physician Health Program ABS-doctors, Royal Dutch Medical Association (RDMA), Utrecht, the Netherlands; 4 Scientific Center for Quality of Healthcare (IQ healthcare), Radboud Institute for Health Sciences, Radboud University Medical Center, Nijmegen, the Netherlands; 5 Tactus Addiction Treatment, Deventer, the Netherlands; 6 Behavioral Science Institute, Radboud University, Nijmegen, the Netherlands; Centre for Addiction and Mental Health, CANADA

## Abstract

**Introduction:**

Substance use disorders (SUD) in physicians often remain concealed for a long time. Peer monitoring and open discussions with colleagues are essential for identifying SUD. However, physicians often feel uncomfortable discussing substance use with a colleague. We explored physicians’ attitudes and norms about substance use (disorders) and their (intended) approach upon a presumption of substance use in a colleague.

**Materials and methods:**

An online cross-sectional survey concerning “Addiction in physicians” was administered by the Royal Dutch Medical Association physician panel. Overall, 1685 physicians (47%) responded. Data were analyzed by logistic regression to explore factors associated with taking action upon a substance use presumption.

**Results:**

Most physicians agreed that SUD can happen to anyone (67%), is not a sign of weakness (78%) and that it is a disease that can be treated (83%). Substance use in a working context was perceived as unacceptable (alcohol at work: 99%, alcohol during a standby duty: 91%, alcohol in the eight hours before work: 77%, and illicit drugs in the eight hours before work: 97%). Almost all respondents (97%) intend to act upon a substance use presumption in a colleague. Of the 29% who ever had this presumption, 65% took actual action. Actual action was associated with male gender and older age (OR = 1.81; 95% CI = 1.20–2.74 and OR = 1.03; 95% CI = 1.01–1.05, respectively).

**Conclusions:**

About one-third of physicians reported experience with a presumption of substance use in a colleague. Whilst most physicians intend to take action upon such a presumption, two-thirds actually do act upon a presumption. To bridge this intention-behavior gap continued medical education on signs and symptoms of SUD and instructions on how to enter a supportive dialogue with a colleague about personal issues, may enhance physicians’ knowledge, confidence, and ethical responsibility to act upon a presumption of substance use or other concerns in a colleague.

## Introduction

Physical illness, mental problems and substance use disorders (SUD) hit physicians as any other individual. SUD is associated with personal harm, but may also contribute to physician impairment; thus influencing quality of health care [1, 2]. Substance use or SUD has been shown to contribute to impairment in 20–40% of these cases [3–5], with consequences as mistakes in diagnosis and medical procedures, or problematic communication skills [6, 7]. Several studies provide additional evidence for self-reported work-related effects of substance use or SUD [8, 9]. It has however been suggested that other variables, like male gender, better predict malpractice and that physicians with mental health problems report medical errors more often due to negative self-appraisal resulting from cognitive bias [10]. Physicians well-being is not only relevant for the individual physician, but also increasingly seen as an important indicator for quality of patient care [2]. However, the impact of substance use among physicians is an understudied area.

The lifetime prevalence of SUD in physicians is, according to American numbers, slightly higher (15.4%) than in the general population (12.6%) [11, 12]. In Europe, hazardous alcohol and drug use among physicians are estimated at 18–23% and 3% respectively [13–19]. Compared to the general population, physicians more often use alcohol and prescription medicines, including sedatives and opioids [20, 21]. Predisposing factors for SUD in physicians include stress and high expectations at work, disrupted life-style due to inconsistent working hours, and easy access to prescription drugs [15, 22, 23].

Normally, society sees physicians as healthy individuals treating patients, not as individuals who might be patients in need of help themselves [24–26]. A qualitative study in New Zealand suggested that this paradox deters physicians to access healthcare for their own health problems [27]. Besides a tendency of minimization and denial of early symptoms [27], impaired physicians indicated that they feel ashamed and that they fear accessing mental healthcare [24, 25]. Their fear includes being stigmatized as a ‘patient’ or ‘addict’ and losing their professional confidentiality and career perspective [24–26, 28]. Minimization and denial of early symptoms and the authority to prescribe drugs subsequently impede identification of SUD in physicians [29].

Seeking help for SUD by physicians often occurs after a pivotal event, such as being caught using substances at work [30]. Some typical signs for SUD in healthcare professionals have been described, including frequent absences, inaccessibility to patients and staff, decreased performance, large quantities of drugs ordered, multiple prescriptions for family members, and vague letters of reference [23, 31]. Since colleagues and other health care professionals may notice these signs earlier than formal agencies, peer support and peer report are important mechanisms for identifying substance use problems in physicians [30, 32].

Although physicians say they feel the ethical duty to report substance use in colleagues [33], several reasons withhold them of reporting a colleague to a Physician Health Program (PHP) [34]. Frequently cited reasons for not reporting impaired colleagues include fear of retribution and excessive punishment of the impaired physician, the assumption that someone else is taking care of the problem, believing it is not your responsibility, believing nothing would happen as result of the report, and not knowing how to report [5, 32, 35]. It is known that attitudes and norms play an important role in intention and actual behavior [36]. However, we do not know in what way attitudes towards SUD and norms about substance use contribute to a physician’s intention and behavior to act upon a substance use presumption in a colleague [17].

A Dutch survey on impairment and incompetence in healthcare professionals (N = 1238; physicians represented 38% of the respondents) revealed that 8.5% of the respondents experienced impairment in a colleague due to substance use during the last year [5]. This Dutch survey and a comparable American survey in physicians revealed that almost three quarters of the respondents (64–72%) think that they know how to deal with impairment or incompetence if present [5, 32]. A similar proportion of the health professionals (66–69%) say they acted upon an actual impairment or incompetence presumption [5, 32], including talking with the colleague, reporting the colleague to the board of the organization or other relevant authority, or discussing the experience with colleagues. These studies did not investigate factors associated with taking actual action upon a presumption of impairment or incompetence [18].

Altogether, substance use among physicians is a highly relevant, but understudied area. Colleagues seem to be aware of substance use and SUD among colleagues, but it is unclear how many actually take action upon a substance use presumption in a colleague. To identify factors that are associated with taking action, we investigated (a) physician’s attitudes towards SUD, (b) their norms about work-related substance use, and (c) their intended and actual actions upon a presumption of substance use in a colleague. We also explored whether physicians’ attitudes, norms and characteristics predicted their actual action upon a substance use presumption.

## Materials and methods

### Design and participants

An online survey concerning “Addiction in physicians” was composed and released by the Royal Dutch Medical Association (RDMA) in September 2016. The survey was administered to an existing physician panel of the RDMA. Through this panel the RDMA aims to efficiently collect information concerning physicians’ opinions regarding specific topics. The panel distributes maximally 8 survey invitations per year and physicians decide to participate per survey. In total, the physician panel of the RDMA consists of 3605 Dutch physicians.

Panelists received an email with the invitation to participate in and the link to the online survey. Panelists had three weeks to complete the survey. They received two reminders to respond to the survey. Encrypted data were collected via the web-based survey platform. This survey data was synthesized with encrypted demographic information of the respondents.

The study was reviewed and approved by the internal ethical review board of the Royal Dutch Medical Association. Participants of the RDMA physician panel were informed about the nature of the survey beforehand and they could decide to participate or not. Data were analyzed anonymously.

### Measures

The survey consisted of 26 closed and 10 open questions on four main themes: attitudes towards SUD, norms about work-related substance use, presumptions of substance use in a colleague at work, and the Dutch PHP ABS-doctors. For this study, we selected 10 questions that were related to the aim of our study, see Box 1.

**Box 1. Theses and questions selected of the RDMA survey.**

**Table:** 

*Attitudes*	A) SUD can happen to anyone
	B) SUD is not a sign of weakness
	C) SUD is a disease that can be treated
*Norms*	D) Do you think you can drink alcohol at work?
	E) Do you think you can drink alcohol during a standby duty?
	F) Do you think you can drink alcohol in the eight hours before work?
	G) Do you think you can use illicit drugs in the eight hours before work?
*Presumptions*	How would you react when you presume a colleague of using alcohol or medicines of the Controlled Substances Act at work?
	Did you ever presume a colleague of using alcohol or medicines of the Controlled Substances Act at work?
	If yes: What did you do?

RDMA = Royal Dutch Medical Association, SUD = Substance Use Disorder.

SUD attitude response categories were “agree”, “don’t know” or “disagree”. Questions regarding physicians’ norms about work-related substance use were assessed by the response categories “yes”, “don’t know”, or “no”. Physicians were asked to indicate what they would do and, if applicable, what they did when presuming a colleague of using alcohol or medicines of the Controlled Substances Act. Response categories included “I would do nothing”/“I wondered how to act, but eventually did nothing”, “I enter(ed) the dialogue with the colleague in question”, “I discuss it with another colleague”, “I discuss(ed) it with the manager”, “I consulted the Dutch PHP and followed their procedures”, “other, namely …”, and “don’t know”.

Available demographical information included gender, age, medical specialty, years in practice, and working situation. Medical specialty was divided into four categories based on differences in SUD attitudes per discipline [37] and convenience with regard to group size: general practice, (psycho) social medicine, contemplative somatic medicine, and surgical and supportive medicine, see S1 Table.

## Statistical analysis

Dichotomous variables were created for the three SUD attitudes theses (“agree” response versus responses “don’t know” + “disagree”), since we were interested in the extent to which physicians reported empathetic attitudes towards SUD. In order to formulate all three SUD attitude theses positively, one of the theses on SUD attitudes was reversed (from “SUD is a sign of weakness” to “SUD is not a sign of weakness”). Dichotomous variables were also created for the four substance use norm questions (“no” response versus responses “don’t know” + “yes”), since our interest was the extent to which physicians saw work-related substance use as unacceptable. Intended and actual approaches to a presumption of substance use in a colleague at work were combined into three categories, in order to reflect the nature of action. These categories were direct action (“I enter(ed) the dialogue with the colleague in question”), indirect action (“I discuss it with another colleague”, “I discuss(ed) it with the manager”, and “I consulted the Dutch PHP and followed their procedures”), and no action (“I would do nothing” and “I wondered how to act, but eventually did nothing” and “don’t know”).

Chi-square tests and Analysis of Variance (ANOVA) tests were used to compare the characteristics of the respondents with the non-respondents. In order to test whether physicians’ attitudes towards SUD and norms about work-related substance use were associated with physician characteristics, Chi-square tests or independent sample t-tests were used when appropriate. Chi-square tests and ANOVA were used to test whether intended and actual approaches upon an substance use presumption in a colleague were associated with physician characteristics. Post hoc Bonferroni tests were conducted to compare attitudes, norms, and approaches by physician characteristics. Binary logistic regression with backward elimination (likelihood ratio test, α = 0.05) was used in order to explore factors among physician characteristics, attitudes, norms that predicted actual action. The characteristics ‘years in practice’ and ‘working situation’ were not included in the regression analysis due to their strong correlation with age. Multicollinearity of data was checked by determining the tolerance and variance inflation factor (VIF). Sensitivity analyses, excluding the retired physicians, were performed in order to verify timeliness of our findings. All statistical analyses were performed using the Statistical Package for Social Sciences (SPSS), version 22.0 for Windows (IBM Corporation, Armonk, NY).

## Results

Of the 3605 participating Dutch physicians, 1685 (47%) completed the survey. Respondents were more often female, older, and had more years in practice than non-respondents, see Table 1. Over half of the (psycho) social medicine group responded upon the survey. The other specialty groups had more non-respondents than respondents. The respondent sample consisted of general practitioners (34%), (psycho)social physicians (28%), contemplative somatic specialists (22%), and supportive and surgical specialists (15%), see Table 1 for a more detailed description of the sample.

**Table 1:** Characteristics of respondents and non-respondents (N = 3605).

Characteristic	All panel participants	Respondents	Non-respondents	
Total *(N (%))*	3605	1685 (47)	1920 (53)	
Gender *(N (%))*				
Male	1818	807 (44)	1011 (56)	*
Female	1727	861 (50)	866 (50)	
Age in years				
*(Mean (SD))*	52 (13)	53 (13)	52 (13)	*
Specialty group *(N (%))*				
General practice	1253	566 (45)	687 (55)	*
(Psycho) social	874	470 (54)	404 (46)	
Contemplative somatic	862	377 (44)	485 (56)	
Surgical and supportive	591	263 (45)	328 (55)	
Years in practice				
*(Mean (SD))*	21 (13)	22 (12)	21 (13)	*
Working situation *(N (%))*				
In training	371	152 (41)	219 (59)	*
Working part time	589	293 (50)	296 (50)	
Working fulltime	2040	951 (47)	1089 (53)	
Retired	471	232 (49)	239 (51)	

N = number, SD = standard deviation

* = p < .05.

### Attitudes towards SUD

The majority of the respondents agreed with the statement that SUD can happen to anyone (67%), that SUD is not a sign of weakness (78%), and that SUD is a disease that can be treated (83%) (Table 2). Agreement to the thesis “SUD can happen to anyone” was significantly associated with a younger age (52 vs 55 years), less years in practice (21 vs 23 years), and the working situation in training (+13 to +18 percentage points compared to working fulltime and retired). Agreement to the thesis “SUD is not a sign of weakness” was associated with female gender (+8 percentage points compared to male gender), a younger age (52 vs to 56 years), specialty group being (psycho)social medicine, general practice, or contemplative somatic medicine (+11 to +14 percentage points compared to surgical and supportive medicine), less years in practice (21 vs 25 years), and the working situations in training and working full time (+11 to +17 percentage points compared to retired). Agreement to the thesis “SUD is a disease that can be treated” was associated with specialty group being (psycho)social medicine (+8 percentage points compared to surgical and supportive medicine) and the working situation in training (+13 percentage points compared to retired).

**Table 2:** Descriptive statistics of substance use disorder attitudes and norms regarding work-related substance use (N = 1685).

	Agreement with the thesis that …			Unacceptability of …			
Characteristics	SUD can happen to anyone^a^	SUD is not a sign of weakness ^b^	SUD is a disease that can be treated ^c^	drinking alcohol at work ^d^	drinking alcohol during a standby duty ^e^	drinking alcohol in eight hours before work ^f^	using illicit drugs in eight hours before work ^g^
Total *(N (%))*	1131 (67)	1313 (78)	1394 (83)	1663 (99)	1529 (91)	1293 (77)	1639 (97)
Gender *(N (%))*							
Male^1^	540 (67)	598 (74)*^2^	657 (81)	791 (98)*^2^	722 (90)	602 (75)*^2^	778 (96)*^2^
Female^2^	581 (68)	703 (82)*^1^	724 (84)	856 (99)*^1^	792 (92)	680 (79)*^1^	845 (98)*^1^
Age in years							
*(Mean (SD))*	52 (13)*	52 (12)*	53 (13)	53 (13)	53 (13)	53 (12)	53 (13)
Specialty group *(N (%))*							
General practice^3^	384 (68)	446 (79)*^6^	462 (82)	563 (100)*^6^	542 (96)*^5^^,^^6^	438 (77)	552 (98)
(Psycho) social^4^	321 (68)	386 (82)*^6^	410 (87)*^6^	466 (99)	446 (95)*^5^^,^^6^	365 (78)	455 (97)
Contemplative somatic^5^	252 (67)	296 (79)*^6^	306 (81)	372 (99)	318 (85)*^3^^,^^4^	293 (78)	369 (98)
Surgical and supportive^6^	168 (64)	178 (68)*^3^^,^^4^^,^^5^	208 (79)*^4^	253 (97)*^3^	215 (82)*^3^^,^^4^	193 (74)	255 (97)
Years in practice							
*(Mean (SD))*	21 (12)*	21 (12)*	23 (13)	22 (12)	22 (12)	22 (12)	22 (12)
Working situation *(N (%))*							
In training^7^	120 (79)*^10^	129 (85)*^10^	135 (89)*^10^	148 (97)	138 (91)	111 (73)	146 (96)
Working part time^8^	198 (68)	229 (78)	245 (84)	289 (99)	270 (92)	218 (74)	286 (98)
Working fulltime^9^	631 (66)*^7^	753 (79)*^10^	785 (83)	941 (99)	855 (90)	751 (79)	929 (98)
Retired^10^	142 (61)*^7^	158 (68)*^7^	176 (76)*^7^	228 (98)	215 (93)	168 (72)	223 (96)

N = number, SD = standard deviation, SUD = Substance Use Disorder

* = p < .05.

^1–10^ Significantly different percentages are printed with corresponding number of the different characteristic.

^a^ Reference: mean age in years = 55 (SD = 12), mean years in practice = 23 (SD = 12).

^b^ Reference: mean age in years = 56 (SD = 13), mean years in practice = 25 (SD = 13).

^c^ Reference: mean age in years = 54 (SD = 12), mean years in practice = 23 (SD = 13).

^d^ Reference: mean age in years = 50 (SD = 14), mean years in practice = 20 (SD = 14).

^e^ Reference: mean age in years = 52 (SD = 12), mean years in practice = 21 (SD = 12).

^f^ Reference: mean age in years = 54 (SD = 13), mean years in practice = 23 (SD = 13).

^g^ Reference: mean age in years = 57 (SD = 11), mean years in practice = 26 (SD = 12).

### Norms about work-related substance use

A vast majority of the respondents considered alcohol at work (99%), alcohol during a standby duty (91%), alcohol or illicit drugs in the eight hours before work unacceptable (77% and 97% respectively), see Table 2. Agreement to the norm that using alcohol at work is unacceptable was associated with female gender (+1 percentage point compared to male gender) and specialty group general practice (+3 percentage points compared to surgical and supportive medicine). Unacceptability of using alcohol during a standby duty was associated with specialty group being general practice or (psycho)social medicine (+10 to +14 percentage points compared to contemplative somatic and surgical and supportive medicine). Unacceptability of using alcohol or illicit drugs in the eight hours before work was associated with female gender (+2 to +4 percentage points compared to male gender).

### Intended and actual action upon a presumption of substance use in a colleague

**Fig 1:**
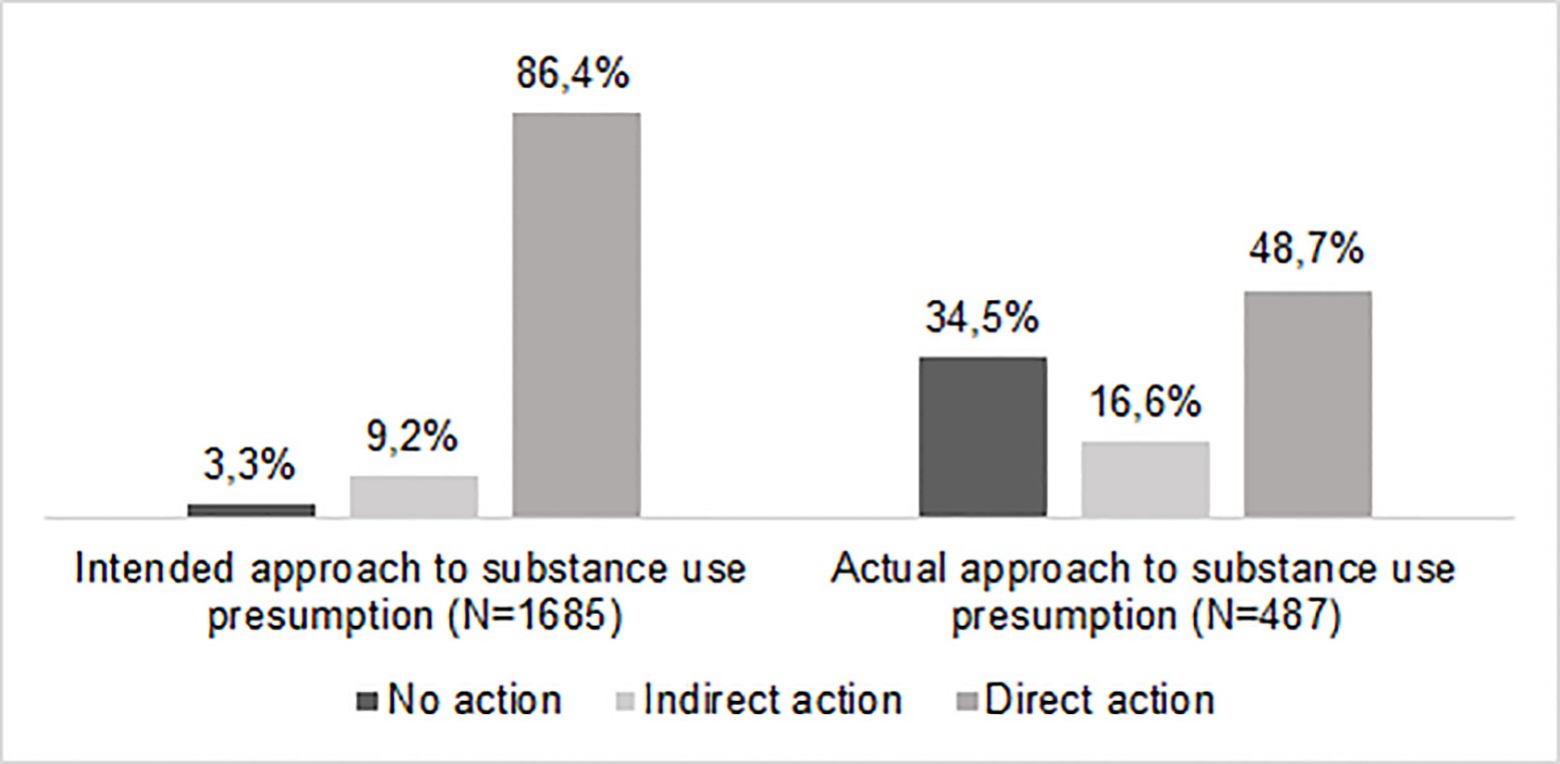
Intended and actual approach upon a presumption of substance use in a colleague.

Almost all physicians (95%) indicated that they would take action upon a substance use presumption in a colleague, either through direct (86%) or indirect (9%) action (Table 3). Approximately three out of ten physicians (N = 487; 29%) answered “yes” to the question “Did you ever presume a colleague of using alcohol or medicines of the Controlled Substances Act at work?” Almost half of them (49%) took direct action (i.e. entered the dialogue with the colleague in question), 17% took indirect action (i.e. discussed the presumption with others), while 34% of the physicians took no action (Table 3). Taking action was more often reported in the hypothetical situation of a substance use presumption in a colleague than as a result of an actual presumption (97% versus 65%) (Fig 1). Male physicians were more likely to enter the dialogue with the colleague in question, whereas female physicians were more likely to do nothing. Being older (+4 to +5 years) and working more years in practice (+3 to +4 years) were associated with higher likeliness of entering the dialogue with the colleague in question or discussing the presumption with others. Retired physicians showed highest rates of taking direct and indirect action.

**Table 3:** Descriptive statistics of intended approach, experience, and actual approach upon a presumption of substance use in a colleague.

	All respondents	Intended approach (N = 1685) ⁺			Ever presumed colleague of substance use^a^	Actual approach (N = 487)			
Characteristics		Direct action	Indirect action	No action		Direct action	Indirect action	No action	
Total *(N (%))*	1685	1455 (86)	155 (9)	56 (3)	487 (29)	237 (49)	81 (17)	168 (34)	
Gender *(N (%))*									
Male^1^	807	708 (88)	70 (9)	22 (3)	270 (33)*^2^	156 (58)	42 (16)	71 (26)	*
Female^2^	861	733 (85)	84 (10)	32 (4)	212 (25)*^1^	80 (38)	38 (18)	94 (44)	
Age in years									
*(Mean (SD))*	53 (13)	53 (12)	53 (13)	52 (14)	56 (11)*	58 (11)	57 (11)	53 (12)	*
Specialty group *(N (%))*									
General practice^3^	566	481 (85)	57 (10)	21 (4)	191 (34)*^5^	95 (50)	34 (18)	62 (32)	
(Psycho) social^4^	470	414 (88)	34 (7)	15 (3)	143 (30)*^5^	73 (51)	21 (15)	49 (34)	
Contemplative somatic^5^	377	328 (87)	37 (10)	8 (2)	78 (21)*^3^^,^^4^	33 (42)	17 (22)	28 (36)	
Surgical and supportive^6^	263	225 (86)	26 (10)	11 (4)	74 (28)	36 (49)	9 (12)	28 (38)	
Years in practice									
*(Mean (SD))*	22 (12)	22 (12)	22 (13)	21 (14)	25 (12)*	26 (11)	25 (12)	22 (12)	*
Working situation *(N (%))*									
In training^7^	152	134 (88)	13 (9)	3 (2)	21 (14)*^9^^,^^10^	8 (38)	4 (19)	9 (43)	*
Working part time^8^	293	249 (85)	26 (9)	15 (5)	66 (23)	19 (29)	11 (17)	36 (55)	
Working fulltime^9^	951	832 (87)	87 (9)	23 (2)	288 (30)*^7^^,^^10^	142 (49)	51 (18)	94 (33)	
Retired^10^	232	193 (83)	23 (10)	12 (5)	98 (42)*^7^^,^^9^	59 (60)	14 (14)	25 (26)	

N = number, SD = standard deviation

* = p < .05.

⁺ There were 19 missings on the intended approach.

^1–10^ Significantly different percentages are printed with corresponding number of the different characteristic.

^a^ Reference: mean age in years = 52 (SD = 13), mean years in practice = 21 (SD = 12).

The full logistic regression model revealed male gender (Odds Ratio (OR) = 2.07; 95%-Confidence Interval (CI) = 1.34–3.21), increasing age (OR = 1.03; 95%-CI = 1.01–1.05), and disagreement with the attitude “SUD can happen to anyone” (OR = .62; 95%-CI = .40-.96) as predictors for taking actual (direct or indirect) action (Table 4). After correction with backward selection, the final logistic regression model revealed male gender (OR = 1.81; 95%-CI = 1.20–2.74) and increasing age (OR = 1.03; 95%-CI = 1.01–1.05) as independent predictors for taking actual action compared to no action (Table 4). In the final logistic regression analysis, physicians’ attitudes and norms did not predict physicians actual approach upon a substance use presumption in a colleague. The models had a moderate discriminative power, with an Area Under the receiver operating characteristics Curve (AUC) of 0.68. There was no indication for multicollinearity (tolerance > 0.8 and VIF < 1.3). The sensitivity analyses excluding the retired physicians did not substantially alter our results (S2 Table).

**Table 4:** Logistic regression of actual action upon a presumption of substance use in a colleague.

	Actual action (N = 473)^α^			
Characteristics	Full logistic regression model ^a^		Final logistic regression model after backward selection ^b^	
Gender *(OR (95% CI))*				
Male	2.07	1.34–3.21*	1.81	1.20–2.74*
Female	ref		ref	
Age in years *(OR (95% CI))*	1.03	1.01–1.05*	1.03	1.01–1.05*
Specialty group *(OR (95% CI))*				
General practice	1.57	.85–2.90	-	
(Psycho) social	1.48	.78–2.82	-	
Contemplative somatic	1.57	.76–3.24	-	
Surgical and supportive	ref		ref	
**Attitudes**				
Agreement with the thesis that … *(OR (95% CI))*				
SUD can happen to anyone	.62	.40-.96*	-	
SUD is not a sign of weakness	1.37	.82–2.30	-	
SUD is a disease that can be treated	1.07	.64–1.79	-	
**Norms**				
Unacceptability of … *(OR (95% CI))*				
Drinking alcohol at work	.63	.13–3.09	-	
Drinking alcohol during a standby duty	1.44	.70–2.93	-	
Drinking alcohol in eight hours before work	1.48	.89–2.44	-	
Using illicit drugs in eight hours before work	1.49	.54–4.10	-	
**Model performance** *(AUC)*	0.68		0.68	

AUC = Area Under the receiver operating characteristics Curve, CI = Confidence Interval, N = number, OR = Odds Ratio, ref = reference category, SUD = Substance Use Disorder

* = p < .05.

^α^ Actual action: direct and indirect action, reference category: no action.

^a^ Constant: beta = -2.215

^b^ Constant: beta = -1.287.

## Discussion

This study aimed to investigate attitudes towards SUD and norms about work-related substance use among physicians, and explored their role in taking action upon a presumption of substance use in a colleague. Overall, physicians showed empathic attitudes towards SUD and their norms regarding work-related substance use were high. Almost one-third of the physicians reported ever having presumed substance use in a colleague at work. Almost two thirds of these physicians took action after such presumption. Male and older physicians were most likely to take action upon a substance use presumption. Attitudes towards SUD and norms about work-related substance use did not predict action upon a substance use presumption in a colleague.

Our study shows that a substantial proportion of physicians ever presumed substance use in a colleague. A survey in 1248 Dutch health professionals reported an experience rate of 2.6% in the past year regarding substance use in a colleague [5]. With an annual rate of nearly 3% it is rather likely that physicians will be confronted with such a presumption at some point of their career [38]. Though our observation of about two thirds of physicians taking action upon such presumption is in line with previous studies among health professionals [5, 32], definitions of taking action vary among studies. In our, and some European studies [5, 17], taking action mainly concerned peer support or informal action (i.e. informal expression of empathic concerns regarding substance use to the colleague in question or discussing how to act upon the presumption with others), whereas American studies commonly describe peer report or formal action (i.e. delating the colleague in question to relevant authorities) [32, 39]. Physicians seem to prefer taking peer support over peer report [40], yet around 40% of physicians and medical students in the United Kingdom, United States and New Zealand indicated that they feel it is not their responsibility to address their colleagues’ mistakes [32, 39–42].

About one third of the physicians in cross-sectional studies reported that they did not act upon impairment or incompetence in a colleague [5, 32]. A frequently cited reason for taking no action was the expectation that someone else would take care of the problem (bystander effect) [5, 32, 35]. Such bystander effect has repeatedly been observed in dangerous and non-dangerous emergencies in different contexts [43], including for instance when witnessing cardiac arrest [44], bullying [45], sexual violence [46], or drug overdose [47]. The decision to take action is explained by the five steps of the bystander or social intervention model [48]. At first, a potential intervener should notice the event, subsequently take it seriously and feel responsibility to intervene, and lastly should know what to do and decide to take action [49]. Besides the bystander effect, a dependency position relative to the colleague in question might affect a physician’s willingness to act [50]. A cohort study in medical students showed that only 13% of the first-year physician-students considers reporting a senior colleague’s mistake, whereas by the end of their medical training, less than 5% is inclined to do so [41].

Most physicians reported empathic attitudes towards SUD and high norms regarding work-related substance use. Female gender, younger age, being in training, and the specialty group psycho-social medicine were associated with slightly more empathic attitudes. Previous studies showed that more contact and familiarity with SUD may contribute to reducing the stigma towards SUD, and development of more empathic attitudes [51, 52]. This can be reached by training in addiction medicine (by for example former substance use impaired health professionals), which showed to be effective in increasing physicians’ knowledge, attitudes, and skills concerning SUD at various academic levels (student, resident, specialist)[53]. Thereby, workplace policy and supervision are suggested to further improve attitudes towards SUD and work-related substance use norms [24].

With regard to work-related substance use norms, male physicians were somewhat more tolerant than female physicians. These results are in line with an American study showing that more than 95% of workers disapproved substance use by a colleague at work [38]. They also showed that disapproval of substance use at work was significantly higher in female workers, compared to male workers. In a US study someone’s disapproval of substance use at work was associated with lower own frequency of substance use before and at work [54]. While female physicians showed higher norms towards work-related substance use, the logistic regression showed that male physicians were more than twice as likely to act in case of a substance use presumption in a colleague. Previous studies have suggested gender differences in empathic ability and moral decision making [55, 56], with females being more resistant to decisions to inflicting physical or moral pain to others [55, 57, 58]. It is tempting to speculate that this might also play a role in delating a colleague in case of a substance use presumption.

Recently, the RDMA published an explicit zero-tolerance policy for substance use by physicians at work [59]. Indeed, an Australian study showed an association between the presence of substance use workplace policies and reduced levels of risky drinking and drug use in workers [60]. Besides policy making, watching your own and your colleagues’ health is essential for optimal patient care. Education programs can raise awareness in physicians about their own health and develop skills to deal with being a patient themselves or when a colleague becomes a patient and requires help [2, 19, 61]. Especially when it comes to SUD, which is often associated with denial [29], peer identification and support by colleagues are important for physicians in order to receive appropriate care at for example mental health facilities or specialized addiction care [30]. A recent Danish study indeed showed that among physicians with unhealthy alcohol use (n = 346), the majority (78%) reported that help seeking is not relevant to them, indicating a low degree of problem recognition [17]. For physicians it is therefore important to know how to identify substance use in a colleague, and how to enter the dialogue when presuming substance use in a colleague [32, 62].

This study should be interpreted in the light of several limitations. Although the response rate for the survey was acceptable, young physicians are underrepresented in our study (less than 16,5% was younger than 40 years). Secondly, response bias cannot be ruled out, which might have led to social desirable answers including an overestimation of empathic attitudes towards SUD, work-related substance use norms, and willingness to take action upon a substance use presumption [63] and/or a selection of respondents with specific attitudes or prior experiences with substance use among colleagues. Thirdly, no validated questionnaires and measures (for example a Likert scale) were used and specialties were grouped partly based on convenience. Due to the broad sample of specialties with small numbers per specialty we were unable to perform analyses at the level of individual specialties.

## Conclusions

About one-third of physicians reported experience with a presumption of substance use or SUD in a colleague. Whilst most physicians intend to take action upon such a presumption, two-thirds actually do act upon a presumption. To bridge this intention-behavior gap continued medical education on signs and symptoms of SUD and instructions on how to express empathic concerns to a colleague about personal issues, may enhance physicians’ knowledge, confidence, and ethical responsibility to act upon a presumption of substance use or other concerns in a colleague. This will ultimately benefit physicians’ health as well as quality of patient care.

## Supporting information

S1 Table Distribution of various medical specialties (N = 1676). (DOCX)

S2 Table Sensitivity analyses: Logistic regression of actual action upon a presumption of substance use in a colleague; sample without retired physicians. (DOCX)
