# Supplementary material for: Physicians' norms and attitudes towards substance use in colleague physicians: A cross-sectional survey in the Netherlands
Source: PLoS One. 2020 Apr 3;15(4):e0231084. doi: 10.1371/journal.pone.0231084 (PMC7122818; doi:10.1371/journal.pone.0231084)
Supplement: S1 Table — (DOCX) [file pone.0231084.s001.docx]

**S1 Table**

| **Specialty group** | | | |
| --- | --- | --- | --- |
| General practice (N=566) | (Psycho) social (N=470) | Contemplative somatic (N=377) | Supportive and surgical (N=263) |
| general practice (N=566) | addiction medicine (N=4) | allergology (N=1) | anesthesiology (N=51) |
|  | forensic medicine (N=7) | cardiology (N=16) | cardiothoracic surgery (N=2) |
|  | infectious disease control (N=2) | dermatology and venereology (N=9) | clinical chemistry (N=2) |
|  | insurance medicine (N=61) | internal medicine (N=74) | clinical genetics (N=3) |
|  | mental disability (N=17) | gastroenterology (N=10) | general surgery (N=40) |
|  | occupational medicine (N=125) | geriatrics (N=144) | medical microbiology (N=9) |
|  | policy and advice (N=10) | neurology (N=24) | neurosurgery (N=3) |
|  | psychiatry (N=124) | pediatrics (N=48) | nuclear medicine (N=5) |
|  | public health (N=68) | pneumology (N=14) | obstetrics and gynecology (N=40) |
|  | social medical assessment and counseling (N=4) | rehabilitation medicine (N=25) | ophthalmology (N=17) |
|  | tuberculosis control (N=1) | rheumatology (N=11) | orthopedics (N=17) |
|  | youth health care (N=47) | sports medicine (N=1) | otolaryngology (N=9) |
|  |  |  | pathology (N=10) |
|  |  |  | plastic surgery (N=6) |
|  |  |  | emergency medicine (N=10) |
|  |  |  | radiology (N=19) |
|  |  |  | radiotherapy (N=9) |
|  |  |  | urology (N=11) |
