# Supplementary material for: Physicians' norms and attitudes towards substance use in colleague physicians: A cross-sectional survey in the Netherlands
Source: PLoS One. 2020 Apr 3;15(4):e0231084. doi: 10.1371/journal.pone.0231084 (PMC7122818; doi:10.1371/journal.pone.0231084)
Supplement: S2 Table — (DOCX) [file pone.0231084.s002.docx]

**S2 Table**

|  |  | | **Actual action (N=375)^α^** | | | | |
| --- | --- | --- | --- | --- | --- | --- | --- |
| **Characteristics** | | | Full logistic regression model ^a^ | |  | Final logistic regression model after backward selection ^b^ | |
| Gender *(OR (95% CI))* | | |  |  |  |  |  |
|  | Male | | 2.04 | 1.27-3.29* |  | 1.79 | 1.14-2.81* |
|  | Female | | ref |  |  | ref |  |
| Age in years  *(OR (95% CI))* | | | 1.04 | 1.01-1.06* |  | 1.04 | 1.01-1.06* |
| Specialty group  *(OR (95% CI))* | | |  |  |  |  |  |
|  | General practice | | 1.21 | .59-2.50 |  | - |  |
|  | (Psycho) social | | 1.23 | .58-2.59 |  | - |  |
|  | Contemplative somatic | | 1.34 | .59-3.07 |  | - |  |
|  | Surgical and supportive | | ref |  |  | ref |  |
| **Attitudes** | | |  |  |  |  |  |
| Agreement with the thesis that … *(OR (95% CI))* | | |  |  |  |  |  |
|  | | SUD can happen to anyone | .64 | .39-1.05 |  | - |  |
|  | | SUD is not a sign of weakness | 1.49 | .82-2.73 |  | - |  |
|  | | SUD is a disease that can be treated | .85 | .47-1.56 |  | - |  |
| **Norms** | | |  |  |  |  |  |
| Unacceptability of … *(OR (95% CI))* | | |  |  |  |  |  |
|  | Drinking alcohol at work | | .98 | .17-5.75 |  | **-** |  |
|  | Drinking alcohol during a standby duty | | 1.72 | .80-3.72 |  | **-** |  |
|  | Drinking alcohol in eight hours before work | | 1.32 | .75-2.32 |  | **-** |  |
|  | Using illicit drugs in eight hours before work | | 1.55 | .53-4.56 |  | **-** |  |
| **Model performance** *(AUC)* | | | 0.66 |  |  | 0.66 |  |

AUC = Area Under the receiver operating characteristics Curve, CI = Confidence Interval, N = number, OR = Odds Ratio, ref = reference category, SUD = Substance Use Disorder, * = p<.05.

^α^ Actual action: direct and indirect action, reference category: no action.

^a^ Constant: beta = -2.771, ^b^ Constant: beta = -1.603.
